# STAIR CLIMB TIME AND FUNCTIONAL POWER ASSOCIATIONS TO MUSCLE FUNCTION AND PHYSICAL PERFORMANCE

**DOI:** 10.1093/geroni/igac059.1195

**Published:** 2022-12-20

**Authors:** Elsa Strotmeyer, Li-Yung Lui, Nancy W Glynn, Adam Santanasto, Stephen Kritchevsky, Peggy Cawthon, Anne Newman, Jane Cauley

**Affiliations:** School of Public Health, University of Pittsburgh, Pittsburgh, Pennsylvania, United States; University of California at San Francisco, San Francisco, California, United States; School of Public Health, University of Pittsburgh, Pittsburgh, Pennsylvania, United States; School of Public Health, University of Pittsburgh, Oakmont, Pennsylvania, United States; Wake Forest School of Medicine, Winston-Salem, North Carolina, United States; California Pacific Medical Center Research Institute, San Francisco, California, United States; University of Pittsburgh, Pittsburgh, Pennsylvania, United States; University of Pittsburgh, Pittsburgh, Pennsylvania, United States

## Abstract

Repeated stair climbing assesses sustained performance and neuromuscular components of movement, including functional muscle power (force*velocity). However, repeated stair climb associations to standard muscle and physical function measures are not established in older adults. We hypothesized that stair climb time (sec), and ascend power (peak and average; Watts=W) over 3 stair climb laps were associated with standard muscle function, physical function and risk factors in the Study of Muscle, Mobility and Aging (SOMMA; preliminary baseline N=455; 76.9+/-5.3 years; 58.0% women; 85.7% White). Adjusting for age, sex, race, and BMI using multivariate linear regression, stair climb time, peak power and average power were significantly associated with all standard muscle (Keiser leg press 1-RM strength and power; grip strength) and physical function (400m walk speed, SPPB and components) measures. Women had worse stair climb performance vs. men (all p< 0.01) including: longer total time (29.6+/-8.1 vs. 27.4+/-6.7 sec), lower peak power (121.9+/-34.5 vs. 161.4+/-39.0 W), and lower average power (94.9+/-24.3 vs. 124.1+/-28.4 W). Adjusting for age, sex, race, BMI, CHAMPS total physical activity/week, CES-D depressive symptoms, and comorbidity count using multivariate linear regression, older age was related to slower stair climb time and lower peak/average power. Other known risk factors were also associated with worse stair climb performance: non-White race (average power only), lower physical activity (peak/average power only), BMI, depressive symptoms, and higher comorbidity (time only). Repeated stair climb time and power may capture unique aspects of functional decline with aging and are associated with standard muscle and physical function measures.

